# Antimicrobial consumption in five adult intensive care units: a 33-month surveillance study

**DOI:** 10.1186/s13756-018-0451-9

**Published:** 2018-12-21

**Authors:** Hanan H. Balkhy, Aiman El-Saed, Ashraf El-Metwally, Yaseen M. Arabi, Sameera M. Aljohany, Muayed Al Zaibag, Salim Baharoon, Adel F. Alothman

**Affiliations:** 1 King Saud bin Abdulaziz University for Health Sciences, Riyadh, Kingdom of Saudi Arabia; 2 Infectious Diseases, King Abdullah International Medical Research Center, Riyadh, Kingdom of Saudi Arabia; 3 Infection Prevention and Control Department, King Abdulaziz Medical City (KAMC), Ministry of National Guard Health Affairs (MNGHA), P.O. Box 22490, Riyadh, 11426 Kingdom of Saudi Arabia; 4 Community Medicine Department, Faculty of Medicine, Mansoura University, Mansoura, Egypt; 5 Intensive Care Department, KAMC, MNGHA, Riyadh, Saudi Arabia; 6 Pathology and Laboratory Medicine Department, KAMC, MNGHA, Riyadh, Saudi Arabia; 7 Cardiac Center & Cardiac Sciences, KAMC, MNGHA, Riyadh, Saudi Arabia; 8 Internal Medicine Department, Infectious Disease Division, KAMC, MNGHA, Riyadh, Saudi Arabia

**Keywords:** Antimicrobial agents, Antimicrobial resistance, Consumption, Defined daily dose, Days of therapy, Saudi Arabia

## Abstract

**Background:**

Estimating the baseline antimicrobial consumption is extremely important to monitor the impact of antimicrobial stewardship activities that aim to reduce the burden and cost of antimicrobial consumption.

**Objectives:**

To quantify service-specific antimicrobial consumption using different metrics.

**Methods:**

A surveillance study was conducted at King Abdulaziz Medical City, Riyadh, Saudi Arabia, between October 2012 and June 2015 in five adult intensive care units (ICUs). Consumption data were collected manually on a daily basis by infection control practitioners. Data were presented as defined daily dose (DDD), days of therapy (DOT) per 1000 patient days, and frequency of daily consumption.

**Results:**

A total of 43,970 DDDs and 46,940 DOTs were monitored during 54,116 patient-days. For the most frequently consumed antimicrobials, the consumption of carbapenems, piperacillin/tazobactam, vancomycin, and colistin (respectively) in all ICUs combined were 255.9, 134.3, 98.2, and 13.6 DDDs per 1000 patient-days and 235.7, 145.9, 129.5, and 117.5 DOTs per 1000 patient-days. For the frequency of daily consumption, carbapenems were the most frequently consumed antimicrobial group in medical/surgical, burn, and step-down ICUs while piperacillin/tazobactam was the most frequently consumed antimicrobial in neuro-surgical and cardio-thoracic ICUs.

**Conclusion:**

High consumption of broad-spectrum antimicrobial agents such as meropenem and piperacillin/tazobactam is observed in multiple ICUs in a tertiary care hospital. Meropenem consumption is considerably higher than similar ICUs internationally. Future studies focusing on concurrent monitoring of antimicrobial resistance and identifying patient and physician characteristics associated with specific prescription patterns may help in improving judicious antimicrobial consumption.

## Background

Antimicrobials are the most frequently misused therapeutic drugs worldwide [1]. Antimicrobial- associated adverse effects are frequently mild, but sometimes can be life-threatening and require emergency care [2, 3]. The widespread consumption of antimicrobial agents especially broad-spectrum ones may reflect the physician’s concern and the need for effective therapy for severely ill patients. Additionally, it cannot be dissociated from the worldwide problem of antimicrobial resistance (AMR) [4–6]. Ecological data have confirmed higher AMR in countries with higher antimicrobial consumption [7]. The global emergence of AMR has increased patient morbidity, mortality, and health care costs. [8]. With the global concern of rising AMR, the World Health Organization (WHO) has established a five-pillar global action plan to be adopted by member states to address the AMR challenge at the national level [9]. Increasing knowledge of antimicrobial consumption and resistance through surveillance is one of those five pillars. Similarly, the strategic plan for combating AMR in Gulf Cooperation Council (GCC) States called for monitoring the trends of the antimicrobial consumption using different metrics [10].

As stewardship programs can be cost-effective in reducing unnecessary consumption of restricted and non-restricted antimicrobials [11, 12], King Abdulaziz Medical City-Riyadh (KAMC-R) established a stepwise stewardship program and created guidelines for antimicrobial consumption. However, data on the amount of antimicrobial consumption at a tertiary care setting is largely lacking, both locally and nationally [14, 15]. Estimating the baseline antimicrobial consumption may serve multiple stewardship purposes; to identify hospital units/wards of highest consumption, to monitor the impact for future interventions, and to feedback prescribing physicians with the prescription patterns. The latter by itself has been proven very beneficial in reducing antimicrobial consumption [13]. Therefore, the objective of this study was to quantify service-specific antimicrobial consumption using different metrics in intensive care settings.

## Methods

### Setting

The study was conducted at KAMC-R, Ministry of National Guard Health Affairs (MNGHA) in Saudi Arabia. This healthcare system is governmentally funded and provides healthcare services for about 750,000 Saudi National Guard soldiers, employees, and their families. KAMC-R is a 1000-bed tertiary care facility with 11 different intensive care units (ICUs, a total of 140 beds) and 36 wards covering almost all specialties. The five adult ICUs included in this study were medical/surgical, neurosurgical, burn, cardiothoracic, and step-down ICUs. Together, they have 60 beds and provide care for approximately 1800 patients per year, staying for approximately 20,000 patient-days per year. The overall bed utilization in the included ICUs was 91% and the average length of stay was 11 days.

The antimicrobial committee at KAMC-R is a multi-disciplinary committee that reviews and approves antimicrobial agents for the hospital formulary, develops and updates the guidelines for antimicrobial consumption, in addition to establishing the antimicrobial stewardship team. However, at the time of the production of this manuscript, regular stewardship rounds were not initiated yet.

### Population

The five operational adult ICUs at KAMC-R at the start of the study were included. For the numerator, all adult patients admitted to one of the included ICUs during the study period and received at least one dose of one of the included antimicrobials. For the denominator data, all adult patients admitted to one of the included ICUs during the study period irrespective of antimicrobial consumption. Exclusion criteria included age < 18 years, consumption of antimicrobials not included in the study, and consumption of antimicrobial by a route other than parenteral or oral routes.

### Study design

A surveillance study was conducted at KAMC-R, Saudi Arabia, between October 2012 and June 2015. The study was approved by the ethical committee of King Abdullah International Medical Research Center (KAIMRC) and was funded by KAIMRC.

### Outcome definition

Defined daily dose (DDD, expressed in grams) was defined as the average maintenance dose per day for a drug used for its main indication in a 70 kg adult, as per the WHO and Anatomical Therapeutic Chemical (ATC) definition of a specific DDD [16]. Days of therapy (DOT) were defined as the sum of days (including admission and discharge days) for which any amount of a specific antimicrobial agent was administered to individual patients [17]. Patient days were calculated as the number of patients who were present for any portion of each day (including admission and discharge days) of a calendar month at a specific ICU. Included antimicrobials were aminoglycosides (amikacin or gentamicin), carbapenems (imipenem or meropenem), cephalosporins (ceftriaxone, cefotaxime, ceftazidime, or cefepime), fluoroquinolones (ciprofloxacin or norfloxacin), piptazocin (piperacillin/tazobactam), vancomycin, tigecycline, colistin, caspofungin, and amphotericin B. The frequency of daily antimicrobial consumption was defined as the number of times a specific antimicrobial was consumed out of all times any antimicrobial was consumed.

### Data collection

Data of antimicrobial consumption were collected prospectively on a daily basis by infection control practitioners, using specially created data entry forms. The following variables were recorded; age, gender, ICU type, name, dose, frequency, and route of antimicrobial consumption. The antimicrobial event was recorded (as a new row in the data file) once a patient received at least one dose of one of the included antimicrobials during a certain day. Each antimicrobial event (row) was considered as one day of therapy. The same patient can contribute to more than one antimicrobial event on the same day if he/she received more than one antimicrobial agent on the same day. The same patient can contribute to more than one specific antimicrobial event during the same admission if he/she received the same antimicrobial for more than one day.

### Statistical methods

Continuous variables were presented as means, standard deviations, and sums. Categorical variables were presented as frequencies and percentages. The amount of DDD consumption was calculated separately for each antimicrobial by dividing the total amount of consumption in grams by antimicrobial-specific average DDD. The amounts of antimicrobial consumption were presented as DDD and DOT per 1000 patient days. Mann–Whitney test and Kruskal Wallis test were used to test significant differences in DDD and DOT by gender and age groups, respectively. All *P*-values were two-tailed. P-value < 0.05 was considered as significant. SPSS (Version 23.0. Armonk, NY: IBM Corp) was used for all statistical analyses.

## Results

Over the 33 months of the study, 43,970 DDDs and 46,940 DOTs were monitored during 4919 admissions contributing to 54,116 patient-days. As shown in Table 1, the majority of the antimicrobial consumption (as sum of DOTs in all ICUs) was observed in males (57.9%), those older than 65 years of age (46.6%), and those admitted to medical/surgical ICU (49.3%) or step-down ICU (24.3%). On average, patients consumed 0.94 ± 0.91 DDDs of one or more antimicrobial agent per day. On average, patients consumed 9.5 DOTs of one or more antimicrobial agents per admission (average 11 days). Heavy antimicrobial consumption (as DOTs per admission) was observed in patients admitted to step-down ICU (18.3), followed by medical/surgical (14.3), burn (9.8), neurosurgical (7.0), and lastly cardiothoracic (2.0) ICUs. The top 5 consumed antimicrobial agents (as sum of DOTs in all ICUs) included meropenem (21.4%), piperacillin/tazobactam (16.8%), vancomycin (14.9%), colistin (13.5%), and caspofungin (8.0%). The least frequently consumed antimicrobial agents (as the sum of DOTs in all ICUs) included cefotaxime (0.2%), amphotericin B (0.8%), cefepime (1.2%), amikacin (1.3%), and ceftazidime (1.3%). Almost all (98.7%) antimicrobial consumption was through intravenous route.

**Table 1:** Overall antimicrobial consumption using different metrics by the patient demographics, ICU type, and antimicrobial type

	Average consumption per day		Sum of consumption during the study				Average DOT per admission
	Grams (mean ± SD)	DDD (mean ± SD)	DDD (Sum)	DDD (%)	DOT (Sum)	DOT (%)	
Overall	3.23 ± 4.82	0.94 ± 0.91	43,970.1	100.0%	46,940	100.0%	9.5
Age							
19–45	3.79 ± 5.44	1.08 ± 1.01	12,706.5	28.9%	11,802	25.1%	
46–65	3.27 ± 4.83	0.93 ± 0.9	12,396.3	28.2%	13,277	28.3%	
> 65	2.9 ± 4.4	0.86 ± 0.85	18,867.3	42.9%	21,861	46.6%	
Gender							
Male	3.45 ± 5.02	0.95 ± 0.88	25,725.8	58.5%	27,188	57.9%	
Female	2.92 ± 4.51	0.92 ± 0.95	18,238.0	41.5%	19,743	42.1%	
Type of ICU							
Medical/surgical	2.86 ± 4.42	0.91 ± 0.9	20,981.9	47.7%	23,150	49.3%	14.3
Neurosurgical	5.31 ± 6.25	1.14 ± 0.8	6285.9	14.3%	5504	11.7%	7.0
Burn	3.36 ± 5.14	0.95 ± 0.75	3632.7	8.3%	3820	8.1%	9.8
Cardiothoracic	5.17 ± 5.87	0.93 ± 0.62	2823.4	6.4%	3050	6.5%	2.0
Step-down	2.41 ± 3.84	0.9 ± 1.07	10,246.3	23.3%	11,416	24.3%	18.3
Antimicrobials							
Amikacin	0.63 ± 0.38	0.63 ± 0.38	385.1	0.9%	607	1.3%	
Gentamicin	0.19 ± 0.13	0.8 ± 0.53	684.5	1.6%	851	1.8%	
Imipenem	1.53 ± 0.62	0.77 ± 0.31	2079.1	4.7%	2710	5.8%	
Meropenem	2.34 ± 1.02	1.17 ± 0.51	11,770.4	26.8%	10,047	21.4%	
Ceftriaxone	2.02 ± 1.11	1.01 ± 0.55	1155.5	2.6%	1142	2.4%	
Cefotaxime	2.89 ± 0.49	0.72 ± 0.12	69.4	0.2%	96	0.2%	
Ceftazidime	3.14 ± 1.81	0.78 ± 0.45	495.0	1.1%	631	1.3%	
Cefepime	2.92 ± 1.53	1.46 ± 0.77	856.2	1.9%	586	1.2%	
Ciprofloxacin, IV	0.72 ± 0.34	1.44 ± 0.67	3974.2	9.0%	2760	5.9%	
Ciprofloxacin, oral	0.76 ± 0.3	0.76 ± 0.3	78.4	0.2%	103	0.2%	
Norfloxacin, IV	0.45 ± 0.46	0.57 ± 0.71	463.6	1.1%	810	1.7%	
Norfloxacin, oral	0.43 ± 0.14	0.54 ± 0.17	19.5	0.0%	36	0.1%	
Piperacillin/ Tazobactam	12.89 ± 4.33	0.92 ± 0.31	7270.2	16.5%	7896	16.8%	
Vancomycin, IV	1.46 ± 0.86	0.73 ± 0.43	4715.5	10.7%	6476	13.8%	
Vancomycin, oral	1.12 ± 0.51	1.12 ± 0.51	596.7	1.4%	533	1.1%	
Tigecycline	0.11 ± 0.05	1.08 ± 0.52	1255.5	2.9%	1163	2.5%	
Colistin	0.35 ± 0.18	0.12 ± 0.06	738.3	1.7%	6360	13.5%	
Caspofungin	0.05 ± 0.02	1.1 ± 0.56	4125.5	9.4%	3742	8.0%	
Amphotericin B	0.29 ± 0.08	8.28 ± 2.28	3237.6	7.4%	391	0.8%	
Route							
Intravenous	3.25 ± 4.84	0.94 ± 0.91	43,329.1	98.5%	46,311	98.7%	
Oral	1.25 ± 1.75	1.02 ± 0.53	641.1	1.5%	629	1.3%	

**Fig. 1:**
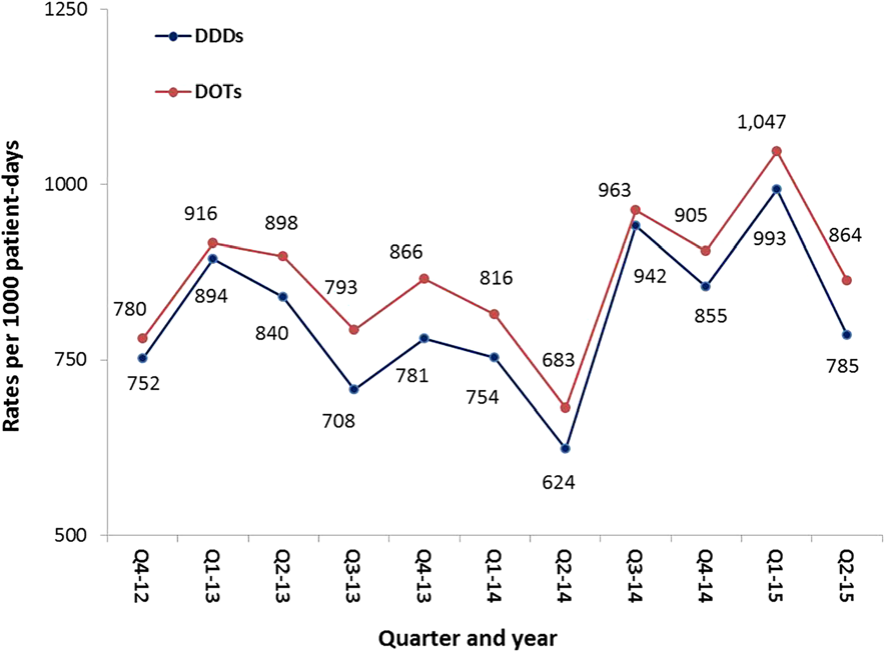
Trends of overall antimicrobial consumption in all adult ICUs, KAMC-R, 2012–2015

As shown in Fig. 1, there was considerable variability in the trend of antimicrobial consumption during the study irrespective of the metrics used. It reached maximum towards the end of 2014 and beginning of 2015 and minimum during the second quarter of 2014. However, the antimicrobial consumption during the first and last quarters of the study was very similar.

Table 2 shows ICU-specific antimicrobial consumption in DDDs per patient-days. For the most frequently consumed antimicrobials, the average amount of carbapenems, piperacillin/tazobactam, and vancomycin in all ICUs combined were 255.9, 134.3, and 98.2 DDDs per 1000 patient-days, respectively. Carbapenems were the most frequently consumed antimicrobial group in all ICUs except cardiothoracic ICU. Piperacillin/tazobactam was the most frequently consumed antimicrobial in cardiothoracic ICU and was preceded only carbapenems in three out of the other four ICUs. Vancomycin was the third to fifth frequently consumed antimicrobials in different ICUs. For the least frequently consumed antimicrobials, the average amount of consumption of colistin, aminoglycosides, and tigecycline in all ICUs combined were 13.6, 19.8, and 23.2 DDDs per 1000 patient-days, respectively. There was no amphotericin B consumption in cardiothoracic ICU.

**Table 2:** ICU-specific antimicrobial consumption in DDDs, KAMC-R,2012–2015

	Medical/ surgical	Neuro-surgical	Burn	Cardio-thoracic	Step-down	Total
Number of DDDs						
Aminoglycosides	449.0	161.4	84.3	24.6	350.2	1069.5
Carbapenems	6890.1	1886.4	1102.5	772.0	3198.6	13,849.5
Cephalosporins	1278.8	610.2	158.3	52.2	476.7	2576.1
Fluoroquinolones	1822.0	635.0	376.5	395.4	1306.8	4535.6
Piperacillin/Tazobactam	3044.2	1528.0	605.8	929.9	1162.4	7270.2
Vancomycin	2362.2	1030.5	513.4	388.0	1018.1	5312.2
Tigecycline	562.5	8.5	324.0	52.5	308.0	1255.5
Colistin	305.6	93.5	138.1	21.4	179.8	738.3
Caspofungin	2572.7	195.4	179.0	187.4	991.0	4125.5
Amphotericin B	1694.9	137.1	150.9	0.0	1254.7	3237.6
Total	20,981.9	6285.9	3632.7	2823.4	10,246.3	43,970.1
Denominator						
Patient days	19,105	7288	6759	7105	13,859	54,116
Admissions	1620	786	391	1499	623	4919
Average length of stay	11.8	9.3	17.3	4.7	22.2	11.0
Rates per 1000 patient-days						
Aminoglycosides	23.5	22.1	12.5	3.5	25.3	19.8
Carbapenems	360.6	258.8	163.1	108.7	230.8	255.9
Cephalosporins	66.9	83.7	23.4	7.4	34.4	47.6
Fluoroquinolones	95.4	87.1	55.7	55.7	94.3	83.8
Piperacillin/Tazobactam	159.3	209.7	89.6	130.9	83.9	134.3
Vancomycin	123.6	141.4	76.0	54.6	73.5	98.2
Tigecycline	29.4	1.2	47.9	7.4	22.2	23.2
Colistin	16.0	12.8	20.4	3.0	13.0	13.6
Caspofungin	134.7	26.8	26.5	26.4	71.5	76.2
Amphotericin B	88.7	18.8	22.3	0.0	90.5	59.8
Total	1098.2	862.5	537.5	397.4	739.3	812.5

**Fig. 2:**
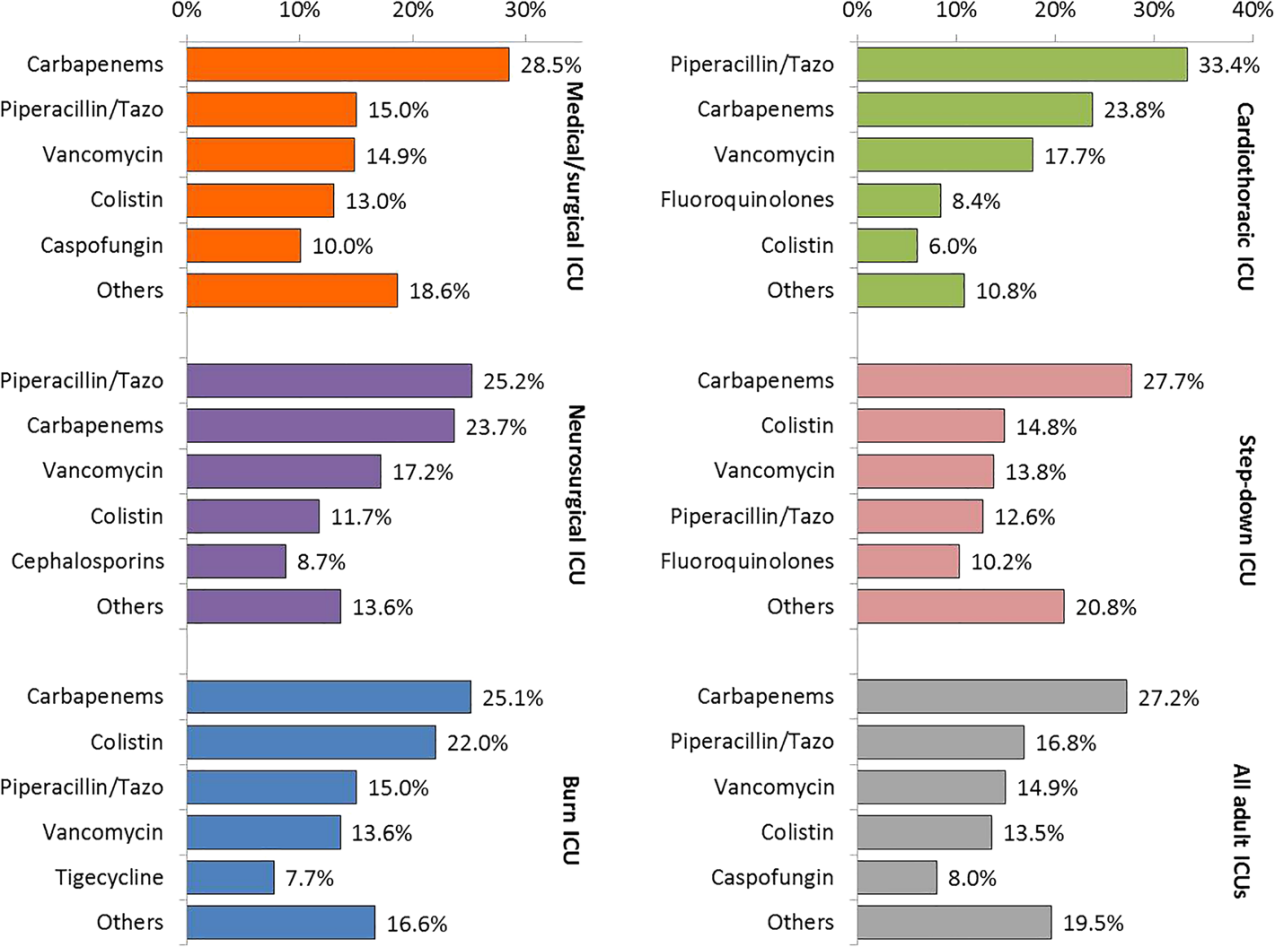
Frequency of daily consumption* of commonly consumed antimicrobials in different adult ICUs, KAMC-R, 2012–2015. Footnote: * Based on DOTs

Table 3 shows ICU-specific antimicrobial consumption in DOTs per patient-days. For the most frequently consumed antimicrobials, the average amount of consumption of carbapenems, piperacillin/tazobactam, vancomycin, and colistin in all ICUs combined were 235.7, 145.9, 129.5, and 117.5 DOTs per 1000 patient-days, respectively. As shown in Table 3 (duration of consumption as DOTs) and Fig. 2, (DOTs-dependent frequency of daily consumption), carbapenems were the most frequently consumed antimicrobial group in medical/surgical, burn, and step-down ICUs while piperacillin/tazobactam was the most frequently consumed antimicrobial in neurosurgical and cardiothoracic ICUs. Colistin was the second most frequently consumed antimicrobial in burn and step-down ICUs while vancomycin was the third most frequently consumed antimicrobial in all ICUs except burn ICU. For the least frequently consumed antimicrobials, the average amount of consumption of amphotericin B and tigecycline in all ICUs combined were 7.2 and 21.5 DOTs per 1000 patient-days, respectively. In all ICUs, the order of the amount (Table 2) and duration (Table 3) of antimicrobial consumption was identical. The consumption data in Tables 2 and 3 were additionally presented as DDDs and DOTs per 100 admissions in supplemental data.

**Table 3:** ICU-specific antimicrobial consumption in DOTs, KAMC-R, 2012–2015

	Medical/ surgical	Neuro-surgical	Burn	Cardio-thoracic	Step-down	Total
Number of DOTs						
Aminoglycosides	683	146	97	55	477	1458
Carbapenems	6609	1302	960	725	3161	12,757
Cephalosporins	1216	480	141	76	542	2455
Fluoroquinolones	1677	399	209	255	1169	3709
Piperacillin/Tazobactam	3473	1387	574	1018	1444	7896
Vancomycin	3438	944	518	539	1570	7009
Tigecycline	512	8	294	53	296	1163
Colistin	3002	642	840	183	1693	6360
Caspofungin	2322	183	167	146	924	3742
Amphotericin B	218	13	20	0	140	391
Total	23,150	5504	3820	3050	11,416	46,940
Rates per 1000 patient-days						
Aminoglycosides	35.7	20.0	14.4	7.7	34.4	26.9
Carbapenems	345.9	178.6	142.0	102.0	228.1	235.7
Cephalosporins	63.6	65.9	20.9	10.7	39.1	45.4
Fluoroquinolones	87.8	54.7	30.9	35.9	84.3	68.5
Piperacillin/Tazobactam	181.8	190.3	84.9	143.3	104.2	145.9
Vancomycin	180.0	129.5	76.6	75.9	113.3	129.5
Tigecycline	26.8	1.1	43.5	7.5	21.4	21.5
Colistin	157.1	88.1	124.3	25.8	122.2	117.5
Caspofungin	121.5	25.1	24.7	20.5	66.7	69.1
Amphotericin B	11.4	1.8	3.0	0.0	10.1	7.2
Total	1211.7	755.2	565.2	429.3	823.7	867.4

## Discussion

ICU-specific antimicrobial consumptions at a major tertiary care hospital were presented in this report using multiple metrics; including DDD, DOT, and the frequency of daily consumption. The calculation of different metrics was essential to improve the clinical and benchmarking usability of this data. For example, it has been shown that the choice of indicator for the surveillance of antimicrobial consumption is critical to evaluate the impact of any stewardship program [15, 18] and to enhance the ability to predict future AMR [19]. Additionally, complementary and sometimes conflicting findings of the impact of antimicrobial stewardship have been reported based on whether DDDs versus DOTs are calculated [15, 18, 20]. This is further complicated by the main stewardship target; the length of stay, number of patients receiving treatment, or duration of treatment [15, 18, 20]. Moreover, DDDs and DOTs are theoretically reflecting different aspects of antimicrobial consumption [21].

The main finding of this study was the high consumption of broad-spectrum antimicrobial agents, specifically carbapenems and piperacillin/tazobactam irrespective of the metrics used. Unfortunately, this study cannot determine the magnitude of inappropriate consumption in the above broad-spectrum antimicrobials. Nevertheless, it has been reported that between 14 and 79% of international antibiotic consumption to treat severe infections in hospitals are inappropriate [22]. Similarly, a recent local study reported that 66% of the consumption of carbapenems and piperacillin/tazobactam in a surgical ward at a tertiary care hospital was either unjustified by culture-test result or done without culture [23]. These rates highlight clinician’s inclination to prescribe broad-spectrum agents. Consistent with current findings, several reports from this hospital [24, 25] and other hospitals in GCC states [26, 27] identified the emergence and/or high burden of carbapenem-resistant Enterobacteriaceae and carbapenem-resistant Acinetobacter. Infections caused by these resistant pathogens are highly fatal and need poly-antimicrobial therapy including carbapenems [28].

Comparison of the consumption rates from this study with local data is impossible or at least difficult. Previous data quantifying antimicrobial consumption at healthcare settings in Saudi Arabia are very limited [14, 15]. For example, none of the published reports have used a prospective design, stratified the consumption by service/location, or used a comprehensive list of antimicrobial groups as shown in this report. On the other hand, the consumption of carbapenems in this study was considerably higher than the rates reported by several reports around the world. For example, it was 255.9 DDDs per 1000 patient-days in current ICUs compared with 36.9 in French ICUs [29], 37.8 in the US National Nosocomial Infections Surveillance (NNIS) medical-surgical ICUs [30], 81.4 in German ICUs [31], 90.0 in the International Nosocomial Infection Control Consortium (INICC) ICUs [32], 58–143 in Swedish ICUs [33], 196.5 in Italian ICUs [34], and 257.1 in Australian & New Zealand ICUs [35].

Unlike carbapenems, the consumption of piperacillin/tazobactam and vancomycin in this study was more comparable to rates reported by above international reports. For example, the consumption of piperacillin/tazobactam in this study was 134.3 DDDs per 1000 patient-days which was similar to Australian & New Zealand ICUs (124.7) [35], higher than NNIS, INICC, and France ICUs (47.2–75.5) [29, 30, 32], and lower than Italian and German ICUs (277.2–412.9) [31, 34]. Similarly, the consumption of vancomycin in this study was 98.2 DDDs per 1000 patient-days which was higher than NNIS, INICC, and German ICUs (36.7–91.9) [30–32] but lower than Italian and Australian & New Zealand ICUs (146.9–191.8) [34, 35].

As most of the DOT-based international reports of antimicrobial consumption were derived from whole hospital data [21, 36] or pediatric/neonatal populations [37, 38], comparing the current ICU-specific DOT rates is challenging. Nevertheless, this study had slightly higher carbapenems (235.7 versus 196.3) but lower piperacillin/tazobactam (145.9 versus 296.3) and vancomycin (129.5 versus 187.2) compared with a study done in an adult ICU in Canada [39]. Fairly similar to this study, piperacillin/tazobactam and/or carbapenems were among the most frequently consumed broad-spectrum antimicrobial agents in Canadian, Australian, and New Zealand ICUs [35, 39]. On the other hand, the majority of international studies show that penicillins [29–31, 34] or cephalosporins [32, 33, 39] are the most frequently consumed antimicrobials in adult ICUs. They represented 30–50% and 26–37% of all antimicrobial consumption in these studies compared with only 16.8 and 5.2% in the current ICUs, respectively.

A major limitation of this study was the inability to address the concurrent appropriateness of antimicrobial consumption. Additionally, the study only targeted antimicrobials frequently consumed in the selected ICU settings, the study does not include less frequently consumed antimicrobials nor those initiated before transfer to the ICU. The target population was National Guard soldiers, employees, and their families at a tertiary care setting. Therefore, interpretation and comparisons of the current findings should be done accordingly. Finally, the study was not designed to take in consideration variations among ICUs in patient mix or predominant bacterial pathogens and their susceptibility patterns. Nevertheless, using the current data to monitor the long-term impacts of different interventions of an antimicrobial stewardship program can potentially help to improve the current prescription practices, reduce cost, and avoid side effects.

## Conclusion

Despite the presence of local written guidelines for antibiotic consumption and the availability of accessible microbiology services, the current finding showed high consumption of broad-spectrum antimicrobial agents such as meropenem and piperacillin/tazobactam in multiple ICUs in a tertiary care hospital. Meropenem consumption was higher than similar ICUs internationally. The findings highlight the urgent need for an effective antimicrobial stewardship program. The GCC Center for infection control has identified AMR as a major threat, and this study represents one of many proposed efforts assisting in mitigating the emergence of AMR in the region [10]. Surveillance and benchmarking of antimicrobial consumption and providing feedback to stakeholders, specifically prescribing physicians can reduce the amount of antimicrobial consumption and probably resistance [13, 40]. Future studies focusing on concurrent monitoring of antimicrobial resistance and identifying patient and physician characteristics associated with specific prescription patterns may help in improving judicious antimicrobial consumption.
